# Supplementary material for: Horizontal transfer generates genetic variation in an asexual pathogen
Source: PeerJ. 2014 Oct 30;2:e650. doi: 10.7717/peerj.650 (PMC4217194; doi:10.7717/peerj.650)
Supplement: Table S1 [file peerj-02-650-s001.pdf]

Supplemental Table S 1: 60 Common type 2 SNPs in strains DVD-3, DVD-31, DVD-161 and DVD-s94

VdLs17 supercontig	Position	REF allele	ALT allele
Supercontig1.8	733056	A	G
Supercontig1.8	733428	C	T
Supercontig1.8	734056	C	T
Supercontig1.8	734471	T	C
Supercontig1.8	734476	G	A
Supercontig1.8	734599	G	A
Supercontig1.8	737029	C	A
Supercontig1.8	737093	C	T
Supercontig1.8	1056983	C	T
Supercontig1.8	1057302	G	A
Supercontig1.9	614488	G	C
Supercontig1.9	615586	G	A
Supercontig1.9	615747	A	G
Supercontig1.9	616120	G	A
Supercontig1.9	1040049	C	T
Supercontig1.9	1041375	C	G
Supercontig1.9	1168734	A	G
Supercontig1.9	1169722	T	C
Supercontig1.9	1170155	G	A
Supercontig1.9	1170336	C	T

Supercontig1.9	1170455	C	T
Supercontig1.9	1170606	C	T
Supercontig1.9	1170827	C	T
Supercontig1.9	1171551	C	T
Supercontig1.9	1172003	C	T
Supercontig1.9	1172039	C	T
Supercontig1.9	1172128	C	T
Supercontig1.9	1175318	C	T
Supercontig1.9	1175597	G	C
Supercontig1.9	1175859	A	T
Supercontig1.9	1176233	T	C
Supercontig1.9	1176250	T	G
Supercontig1.9	1176271	T	C
Supercontig1.9	1176939	C	T
Supercontig1.9	1177851	A	T
Supercontig1.9	1179547	T	C
Supercontig1.9	1181721	A	G
Supercontig1.9	1182314	G	A
Supercontig1.9	1183438	G	T
Supercontig1.9	1185255	C	T

Supercontig1.9	1186010	A	G
Supercontig1.9	1186019	G	A
Supercontig1.9	1186555	T	G
Supercontig1.9	1187295	C	T
Supercontig1.9	1187367	A	T
Supercontig1.9	1187368	G	A
Supercontig1.9	1187548	C	A
Supercontig1.9	1188805	T	A
Supercontig1.9	1189251	C	G
Supercontig1.9	1189970	T	C
Supercontig1.9	1190047	G	A
Supercontig1.9	1190872	A	T
Supercontig1.9	1190909	G	A
Supercontig1.9	1190983	C	T
Supercontig1.9	1191327	T	C
Supercontig1.9	1191388	C	G
Supercontig1.9	1207938	G	A
Supercontig1.9	1207967	G	A
Supercontig1.9	1208061	G	A
Supercontig1.9	1208190	G	A
